# Association between antibodies against group B *Streptococcus* surface proteins and recto-vaginal colonisation during pregnancy

**DOI:** 10.1038/s41598-017-16757-9

**Published:** 2017-11-28

**Authors:** Sonwabile Dzanibe, Gaurav Kwatra, Peter V. Adrian, Sheila Z. Kimaro-Mlacha, Clare L. Cutland, Shabir A. Madhi

**Affiliations:** 1 Department of Science and Technology/National Research Foundation: Vaccine Preventable Diseases, University of the Witwatersrand, Faculty of Health Sciences, Johannesburg, South Africa; 2 Medical Research Council: Respiratory and Meningeal Pathogens Research Unit, Faculty of Health Sciences, University of the Witwatersrand, Johannesburg, South Africa; 3 National Institute for Communicable Diseases: Division of National Health Laboratory Services, Centre for Vaccines and Immunization, Johannesburg, South Africa

## Abstract

Group B *Streptococcus* (GBS) recto-vaginal colonisation in pregnant women is the major risk factor for early-onset invasive GBS disease in their newborns. We aimed to determine the association between serum antibody levels against 11 GBS surface proteins and recto-vaginal acquisition of GBS colonisation during pregnancy. Sera collected from pregnant women at 20–25 weeks and ≥37 weeks of gestation age were measured for IgG titres against GBS surface proteins using  a multiplex immunoassay. Women were evaluated for recto-vaginal colonisation every 4–5 weeks. We observed that the likelihood of becoming colonised with GBS during pregnancy was lower in women with IgG titres ≥200 U/mL against gbs0233 (adjusted OR = 0.47 [95% CI: 0.25–0.89], p = 0.021) and ≥85 U/mL for gbs1539 (adjusted OR = 0.44 [95% CI: 0.24–0.82], p = 0.01) when comparing between women who acquired GBS colonisation and those that remained free of GBS colonisation throughout pregnancy. IgG titres (U/mL) specific to BibA and Sip were higher in pregnant women colonised with GBS (380.19 and 223.87, respectively) compared to women with negative GBS cultures (234.42 and 186.21, respectively; p < 0.01) at ≥37 weeks gestation. Antibodies induced by gbs0233 and gbs1539 were associated with a reduced likelihood of recto-vaginal GBS acquisition during pregnancy and warrant further investigation as vaccine targets.

## Introduction

Group B *Streptococcus* (GBS) is a leading cause of invasive bacterial disease in the first seven days of life (i.e. early-onset diseases; EOD)^1^, with 90% of the cases occurring within the first 24 hours of life^2,3^. Recto-vaginal GBS colonisation during pregnancy is the strongest independent risk factor associated with EOD, in which colonised pregnant women vertically transmit GBS to their newborns either *in utero* or intrapartum. About 10–30% of women are colonised with GBS in the gastrointestinal and genitourinary tract, with colonisation occurring in an intermittent, transient and persistent manner during pregnancy^4–6^. GBS colonisation in the genitourinary tract of women also causes clinical and subclinical acute infections, including chorioamnionitis, endometritis and urinary tract infections^7^. Moreover, GBS colonisation during pregnancy is associated with, late miscarriages, premature birth and stillbirths^8^.

Intrapartum antibiotic prophylaxis (IAP) treatment of women colonised with GBS at 35–37 weeks of gestation age is the recommended strategy to reduce vertical transmission. Since the adoption of IAP treatment in high-income settings, the number of reported EOD cases has declined by 80%^9,10^, however, such a strategy remains logistically challenging and unlikely to be cost-effective for low-income countries, including in settings where 40–60% of deliveries occur outside of health-care settings^11,12^.

Alternative strategies to prevent EOD and other GBS related pregnancy complications are being studied, such as maternal immunisation during pregnancy using GBS capsular polysaccharides (CPS) conjugated to carrier proteins^13–16^. This is primarily based on the observation that neonates born to mothers with low CPS specific antibodies are at a higher risk of developing invasive GBS disease^17,18^.

Recently, molecular characterisation of GBS has revealed the occurrence of capsular switching from the dominant disease causing serotype III to serotype-IV, which could impact on the overall efficacy of investigational serotype-specific polysaccharide based vaccines^19,20^. Therefore, other GBS virulence factors are being evaluated as candidates for vaccine development. Several surface proteins such as the C proteins, BibA, Sip, and pilus island proteins have been reported to confer protection against GBS disease in murine models^21–24^. Furthermore, studies have demonstrated that conjugating CPS to GBS surface proteins results in serotype-independent protection^25–27^. Also, natural induced α-C protein antibodies from pregnant women were shown to be capable of inducing opsonophagocytic killing^28^. Fabbrini *et al*. demonstrated that infants born to mothers with higher antibodies against GBS pilus proteins were associated with reduced risk of developing invasive GBS disease^29^, although, this was not corroborated in an earlier study from South Africa^30^.

A vaccine formulation which includes highly immunogenic GBS surface proteins capable of preventing GBS recto-vaginal colonisation, could reduce the risk of GBS acquisition in pregnant women. This could prevent vertical transmission to their foetuses and newborns, reducing adverse GBS foetal outcomes and invasive disease amongst newborns, as well as protect the women themselves.

The objective of this study was to determine the association of natural induced antibodies to eleven GBS surface proteins and susceptibility to GBS recto-vaginal colonisation from 20 to ≥37 weeks of gestational age.

## Results

Among the 661 pregnant women enrolled, 505 (76.4%) attended all four study visits and were included in the analysis. The main reasons for non-inclusion of the other women were relocation, withdrawal of consent or lost to follow up. The full demographic characteristics of the study cohort are described elsewhere^5^. Overall, GBS colonisation was detected in 161/505 (31.9%) women at 20–25 weeks and in 140/505 (27.7%) at ≥37 weeks of gestation as detailed in Supplementary Table S1. The predominant colonising serotypes were type-Ia (44.1% and 32.9%) and III (32.3% and 35.0%) at 20–25 and ≥37 weeks of gestation age, respectively^5^. Median age, parity, gravidity and number of previous miscarriages did not vary between the “non-colonised” and GBS colonised pregnant women, Supplementary Table S1.

Of the 505 women included in the analysis, 255 (50.4%) were grouped as “non-carriers” and 54 (10.7%) as “new acquisition”. Sixty nine (13.8%) had positive GBS cultured at all study visits (persistently-colonised), 75 (14.8%) were “transiently-colonised” and the remaining 52 (10.3%) were “intermittently-colonised”. Among the women who newly acquired GBS colonisation, 25.9% (14/54) acquired GBS colonisation in the rectal tract only, 24.1% (13/54) in vagina tract only and 50.0% (27/54) had concurrent GBS colonisation in the rectal and vaginal tracts (“recto-vaginal tracts”); Supplmentary Figure S1.

### Association between IgG titres and concurrent GBS colonisation

At 20–25 weeks of gestation, the median IgG titres (U/mL) for BibA protein of GBS NEM316 strain were higher in colonised compared to non-colonised women (398 vs 295; *p* = 0.03), Table 1. A similar trend was observed at ≥37 weeks of gestation age with elevated median IgG titres (U/mL) in colonised compared to non-colonised women for BibA-COH1 (309 vs 245, *p* = 0.01), BibA-NEM316 (380 vs 234, *p* = 0.03), and Sip (224 vs 186, *p* = 0.01). These differences were, however, statistically significant only for BibA-NEM316 (*p* = 0.001) and Sip (*p* = 0.001) after adjusting for cofactor variables, Table 1.

**Table 1:** Cross-sectional comparison of median IgG antibody titres (U/ml) between non-colonised and colonised pregnant women at 20–25 and ≥37 weeks of gestation age.

	Protein	20–25 weeks				37–40 weeks			
		Median IgG (IQR)				Median IgG titre (IQR)			
		Non-colonised (n = 344)	Colonised (n = 161)	*p*-value	Adj *p*-value^α^	Non-colonised (n = 365)	Colonised (n = 140)	*p*-value	Adj *p*-value^α^
Lipoprotein	Gbs0233	169.82 (91.20–389.05)	208.93 (104.71–371.54)	0.21	0.10	131.83 (81.28–309.03)	138.04 (81.28–234.42)	0.90	0.90
	Gbs2106	263.03 (128.82–524.81)	288.40 (131.83–478.63)	0.92	0.90	204.17 (91.20–398.11)	251.19 (147.91–363.08)	0.17	0.17
	Foldase PsrA	213.80 (104.71–489.78)	218.78 (117.49–457.09)	0.84	0.66	173.78 (87.10–363.08)	194.98 (100.00–380.19)	0.72	0.25
Cell wall anchored proteins	FbsA	104.71 (33.88–416.87)	93.33 (34.67–389.05)	0.81	0.80	83.18 (36.31–295.12)	85.11 (38.02–346.74)	0.77	0.97
	BibA-COH1	288.40 (138.04–524.81)	331.13 (169.82–575.44)	0.05	0.57	245.47 (125.89–446.68)	309.03 (165.96–602.56)	0.01	0.24
	BibA-NEM316	295.12 (120.23–630.96)	398.11 (190.55–691.83)	0.03	0.21	234.42 (104.71–478.63)	380.19 (173.78–616.60)	0.03	0.001
	Gbs0393	316.23 (158.49–645.65)	354.81 (194.98–616.60)	0.35	0.62	281.84 (141.25–562.34)	245.47 (128.82–489.78)	0.19	0.10
	Sip	239.88 (120.23–426.58)	257.04 (134.90–407.38)	0.68	0.53	186.21 (87.10–316.23)	223.87 (123.03–354.81)	0.01	0.001
	Gbs1539	181.97 (75.86–446.68)	213.80 (89.13–467.74)	0.45	0.37	147.91 (69.18–363.08)	151.36 (75.86–380.19)	0.84	0.77
	Gbs1356	269.15 (144.54–478.63)	295.12 (173.78–478.63)	0.55	0.80	213.80 (109.65–389.05)	218.78 (95.50–501.19)	0.66	0.77
	Gbs0392	245.47 (107.15–537.03)	269.15 (141.25–501.19)	0.40	0.73	186.21 (95.50–436.52)	213.80 (95.50–380.19)	0.96	0.96

α, p value was adjusted for colonising serotype, maternal age, gestational age, parity and gravity.

IgG titres stratified according to the site at which GBS colonisation was detected showed that antibody levels were associated with the site of colonisation. At 20–25 weeks of gestation age, median IgG titres specific to BibA-NEM316 were significantly higher in women who were colonised in the rectum only (490 U/mL) compared to those who were non-colonised (170 U/mL, *p* = 0.003 adjusted for cofactor variables), Supplementary Figure S2. Women who were colonised with GBS in the recto-vaginal tracts at 20–25 weeks of gestation age had higher median Sip antibody titres (U/mL) compared to women who were colonised only in the rectum (309 vs 204, *p* = 0.018). IgG titres for the remaining GBS proteins were similar between non-colonised women and those who were GBS colonised at the rectal and/or vaginal tract, Supplementary Figure S2.

At ≥37 weeks of gestation age, median IgG titres were higher in women who were colonised in the vaginal tract only compared to non-colonised women for proteins FbsA (169.82 vs 83.18, *p* = 0.015), BibA-NEM316 (478.63 vs 234.42, *p* = 0.001), Foldase (269.15 vs 173.78, *p* = 0.034), Sip (275.42 vs 186.21, *p* = 0.023) and gbs1539 (281.84 vs 147.91, *p* = 0.014). After adjusting for cofactor variables these results remained statistically significant for proteins BibA-NEM316 (p = 0.006) and gbs1539 (*p* = 0.031), Supplementary Figure S3. Protein specific IgG titres were not dependent on the colonising serotype at both 20–25 and ≥37 weeks of gestation age.

Multivairate quantile regression analysis adjusted for cofactor variables showed that GBS colonisation in the vaginal tract alone was associated with higher IgG titres (U/mL) compared to women with only rectal colonisation for proteins Foldase (269.15 vs 91.20, *p* = 0.013), gbs0233(177.83 vs 123.03, *p* = 0.031) and gbs1539 (281.84 vs 114.82, *p* = 0.001) at ≥37 weeks of gestation age, Supplementary Figure S3. Moreover, women with only vaginal colonisation had higher median IgG tires compared to women who were concurrently colonised in the rectal and vaginal tract for proteins FbsA (169,82 vs 69.18, *p* = 0.021) and BibA-NEM316 (478,63 vs 316,23, *p* = 0.029), Supplementary Figure S3.

### Effect of GBS acquisition on protein specific antibody response

To determine the association of naturally occurring GBS surface protein IgG antibodies and the risk of recto-vaginal GBS acquisition during pregnancy, antibody titres were compared between women who were “non-carriers” (255/505, 50.4%) throughout and women grouped as “new-acquisition” cases (54/505, 10.7%).

**Figure 1:**
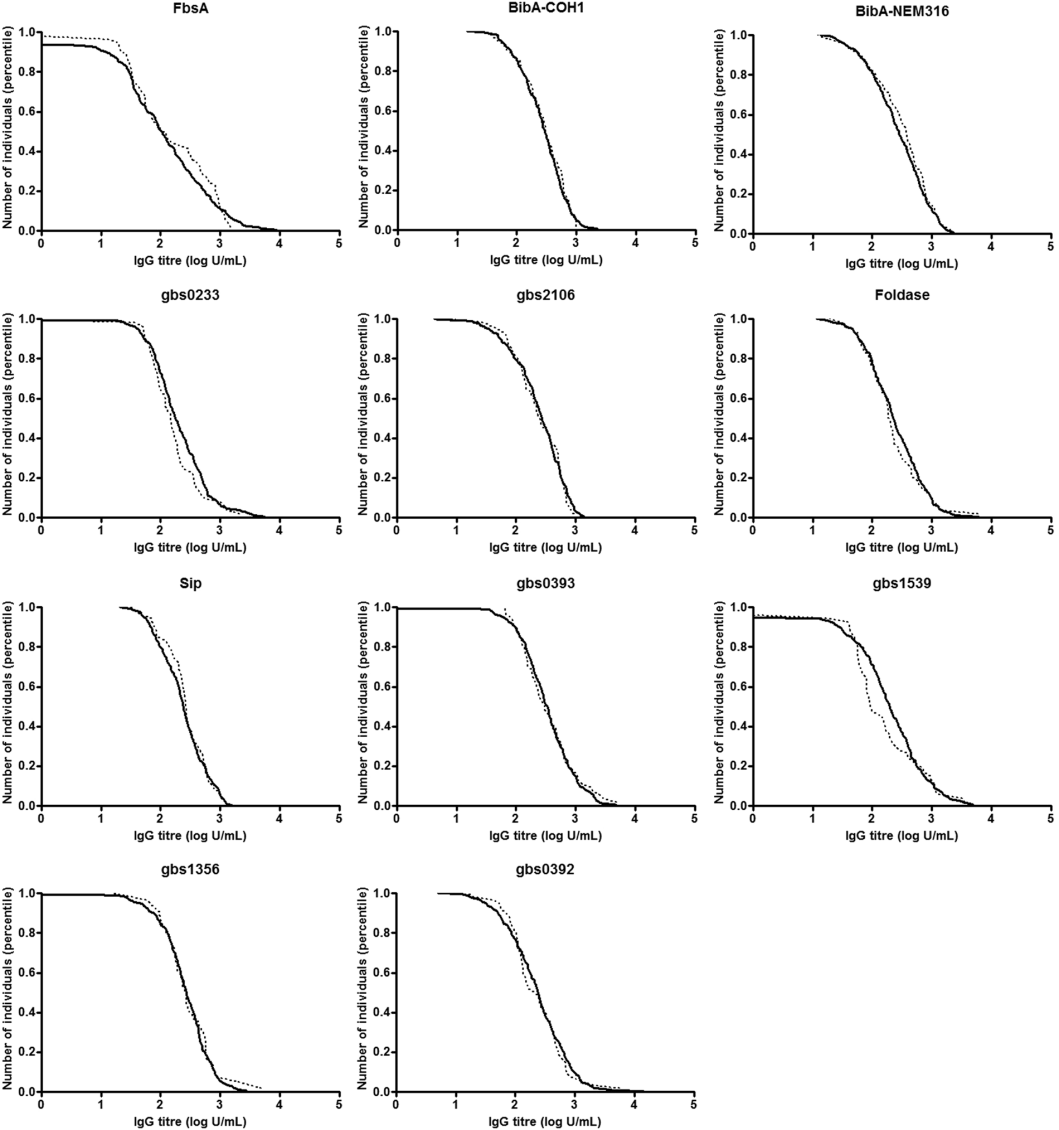
Reverse cumulative plots comparing IgG titres against group B Streptococcus (GBS) surface proteins between women who remained non-colonised throughout pregnancy (solid line) and those who were non-colonised at 20–25 weeks of gestation age but subsequently acquired GBS colonisation (dotted line).

Using reverse cumulative plots, pregnant women who were classified as “non-carriers” had higher IgG titres at enrolment against proteins gbs0233 and gbs1539 compared to the “new-acquisition” group, Fig. 1. Logistic regression analysis was used to determine the IgG antibody titre threshold against gbs0233 and gbs1539 associated with reduced odds of GBS acquisition during pregnancy. Of the “non-carriers”, 46.7% (119/255) had IgG titres ≥ 200 U/mL against surface protein gbs0233 at baseline compared to 29.6% (16/54) of the “new acquisition” group. (adjusted OR = 0.47 [95%CI: 0.25–0.89], *p* = 0.021), Table 2. This strength of association remained similar at a higher threshold, albeit not significant.

**Table 2:** IgG titres against group B Streptococcus (GBS) surface proteins gbs0233 and gbs1539 associated with prevention of acquisition of GBS colonisation.

IgG levels (U/mL)	Non-colonised (n = 255)	New acquisition (n = 54)	OR (95% CI)	*P* value	OR (95% CI)^α^	*P* value^α^
Gbs0233						
≥100	185 (72.5%)	34 (63.0%)	0.64 (0.35–1.19)	0.16	0.60 (0.32–1.14)	0.12
≥200	119 (46.7%)	16 (29.6%)	0.48 (0.26–0.91)	0.022	0.47 (0.25–0.89)	0.021
≥300	85 (33.3%)	12 (22.2%)	0.57 (0.29–1.14)	0.11	0.59 (0.29–1.19)	0.14
≥400	66 (25.9%)	8 (14.8%)	0.50 (0.22–1.11)	0.083	0.53 (0.23–1.18)	0.12
≥500	51 (20.0%)	5 (9.3%)	0.41 (0.15–1.08)	0.063	0.43 (0.16–1.15)	0.092
Gbs1539						
≥85	192 (75.3%)	30 (55.5%)	0.41 (0.22–0.75)	0.003	0.44 (0.24–0.82)	0.01
≥100	181 (71.0%)	26 (48.1%)	0.38 (0.21–0.69)	0.001	0.41 (0.22–0.75)	0.004
≥200	124 (48.6%)	18 (33.3%)	0.53 (0.29–0.98)	0.04	0.5 (0.29–1.00)	0.051
≥300	97 (38.0%)	15 (27.8%)	0.63 (0.33–1.200	0.15	0.64 (0.33–1.22)	0.18

α, adjusted for maternal age, gestational age, parity and gravidit.

Similarly, for gbs1539, the “non-carriers” were 56% more likely to have IgG titres ≥85 U/mL at baseline compared to the “new-acquisition” group (adjusted OR = 0.44 [95% CI: 0.24–0.82], *p* = 0.01). This strength of association between higher gbs1539 antibodies and risk of GBS acquisition persisted at higher thresholds of up to ≥300, albeit not significant thereafter, Table 2. No such association was found for the other 9 GBS surface proteins.

**Figure 2:**
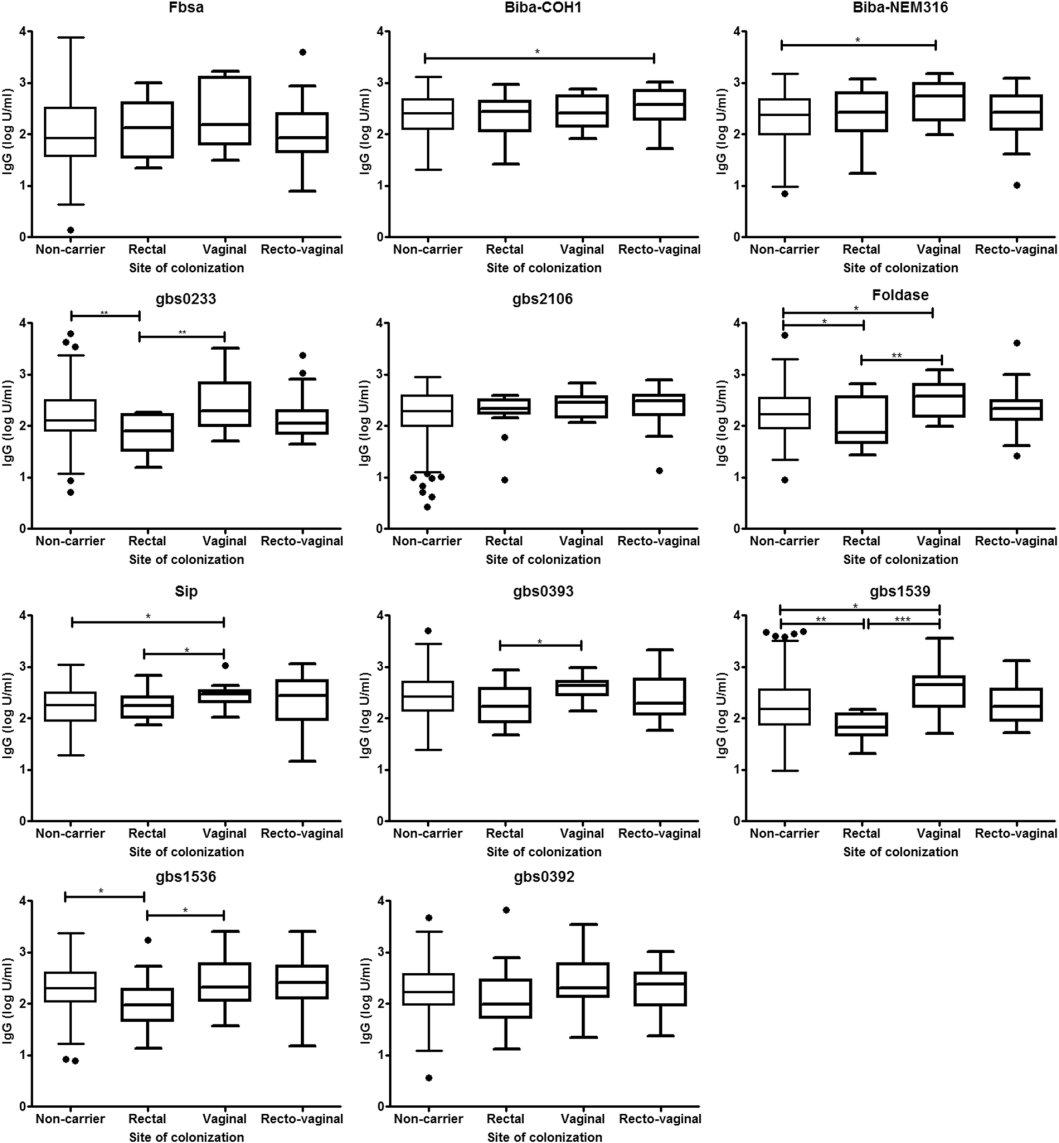
Comparison of median IgG titres to group B *Streptococcus* (GBS) proteins between pregnant women who were non-carriers and those who newly acquired GBS colonisation either at the rectal and/or vaginal tract at ≥37 weeks of gestation age. *p < 0.05, **p < 0.01 and **p < 0.001. Turkey box-and-whisker plots representing the median (line within the box), 25^th^ and 75^th^ percentiles (box), the 1.5 times interquartile distances from the 25^th^ and 75^th^ centiles (whiskers) and outliers (solid dots).

Serum antibodies against GBS surface proteins were dependent on the site where acquisition of GBS colonisation occured. At ≥37 weeks of gestation age, pregnant women who acquired GBS colonisation in the vaginal tract only had higher median IgG titres (U/mL) compared to women who were “non-carriers” for BibA-NEM316 (478.63 vs 234.42, *p* = 0.012), Foldase (269.15 vs173.78, *p* = 0.036), Sip (275.42 vs 186.21, *p* = 0.030) and gbs 1539 (281.84 vs 147.91, *p* = 0.049), Fig. 2. In contrast, lower IgG titres (U/mL) were observed in women who newly acquired GBS colonisation at the rectal tract only at ≥37 weeks gestational age compared to “non-carriers”, including for Foldase (128.82 vs 173.78, *p* = 0.043), gbs0233 (123.03 vs 131.83, *p* = 0.010), gbs1539 (114.82 vs 147.91, *p* = 0.002) and gbs1356 (177.83 vs 213.80, *p* = 0.019), Fig. 2.

Moreover, women who only acquired GBS in the vagina had higher median IgG titres compared to those who acquired GBS colonisation only in the rectum for proteins Sip (*p* = 0.049), Foldase (*p* = 0.0094), gbs0233 (*p* = 0.0039), gbs0393 (*p* = 0.027), gbs1539 (*p* = 0.0004) and gbs1356 (*p* = 0.039); Fig. 2. BibA-COH1 specific median IgG titres (380.19 U/mL) where higher in women who newly acquired GBS colonisation in the recto-vaginal tracts compared to those who were “non-carriers” (257.04, p = 0.041). There was no significant difference observed for the other proteins when comparing protein specific IgG titres in women who acquired GBS colonisation at the recto-vaginal tracts and those who acquired GBS colonisation either in the rectal or vaginal tract only, Fig. 2.

## Discussion

We report here the effects of natural induced antibodies against GBS surface proteins on the dynamics of GBS colonisation during pregnancy in a longitudinal study. Antibodies against specific GBS surface proteins were detected throughout pregnancy irrespective of the women’s colonisation status and the colonising serotype. This observation is attributed to the high GBS acquisition rate of 25.9% that occurred from ≥20 weeks of gestation age onward during the study period^5^. High prevalence of GBS colonisation during pregnancy has also been reported in other studies, being highest in African countries^31^. The protein antibody levels detected in our study were specifically influenced by the site of acquisition of GBS colonisation and gestational age.

Humoral immunity in pregnant women with GBS colonisation has been extensively described for capsular polysaccharides, with serotype specific antibodies significantly increased in women colonised with homotypic CPS serotype^29,32,33^. Similarly, antibody titres against Sip and pilus island proteins have been reported to be higher among colonised pregnant women^29,34^. In our study, cross-sectional analysis at 20–25 and ≥37 weeks of gestation revealed that IgG titres for BibA and Sip proteins were significantly increased in GBS colonised compared to non-colonised pregnant women. Given that the colonising GBS isolates were not evaluated for expressing the investigated surface proteins, the reported antibody titres may be an under estimate of the protein’s capacity to induce natural antibody response. Nevertheless, these proteins are highly conserved among GBS strains and demonstrate a capacity to induce serotype independent immunity^22,23^.

Changes in sera IgG titres against GBS surface proteins with advancing gestation age were dependent on the site where GBS colonisation was detected. IgG titres against select GBS proteins were significantly lower in pregnant women colonised in the rectal tract compared to those who were only colonised at the vaginal tract at ≥37 weeks of gestation. Furthermore, acquisition of GBS at the vaginal tract induced elevated serum IgG titres against select GBS surface proteins. This resulted in significantly lower serum IgG titres against select GBS surface proteins in women who newly acquired GBS colonisation at the rectal tract compared to those that acquired GBS colonisation at the vaginal tract. An increase in systemic antibody production has been reported in women where the vaginal tract was used as the route for immunisation^35,36^. We hypothesize that differential protein expression may occur between the two colonisation sites resulting in an up-regulation of specific proteins and subsequently inducing a higher immune response. Alternatively, the lack of serum antibody stimulation observed following colonisation of the rectal tract may be due to either local immune dependent suppression of colonisation or immunological naivety in the rectum that allows for persistent GBS colonisation. Therefore, future studies are required to investigate GBS protein specific antibodies produced at the mucosal interface together with the transcriptomic profile of GBS depending on the site where colonisation occurs. This information may highlight antigens involved at different pathogenic stages of GBS and thus potential vaccine targets.

The function of naturally induced antibodies was inferred by comparing pregnant women who remained non-colonised throughout the study visits with those that newly acquired GBS after 20–25 weeks of gestation age. Women with IgG titres ≥200 U/mL against gbs0233 were 53% less likely to acquire GBS colonisation. Similarly, having IgG titres ≥85 U/mL against gbs1539 reduced the odds of being colonised by 66%. These results suggest that increasing maternal antibodies to these proteins by vaccination could reduce GBS acquisition and subsequently lower vertical transmission to their respective foetus/newborns. Similar observations were reported for antibodies against CPS of serotype Ia and III, with increasing IgG concentrations associated with reduced odds of acquiring GBS colonisation^37^. Therefore, we reserve that the observed association in this study may be an indirect effect of CPS specific antibodies or other cross-reactive virulence factors not included in this study. Moreover, the expression of the investigated proteins by the GBS isolates recovered from the study participants were not assessed, and thus further limits definite deduction of the protein specific immune response in preventing GBS acquisition.

Immunisation against GBS colonisation during pregnancy has the potential to reduce vertical transmission of the bacterium from mother to foetus/newborns, hence decreasing invasive GBS disease among neonates. The prevention of GBS colonisation will require a vaccine candidate capable of inducing neutralising antibodies at both the mucosal and systemic components^35^. Identifying and targeting antigens that are preferentially expressed for mediating pathogenesis: those having dual importance in colonisation and invasive disease, may increase the efficacy of the vaccine against invasive disease. Moreover, these antigens may be highly expressed and more favourable for immune recognition for prevention of GBS colonisation. Grifantini *et al*. identified 5 genes upregulated during *Neisseria meningitidis* adhesion, two of which were putative proteins, using DNA-microarray that were capable of inducing antibodies with opsonophagocytic activity in mice^38^. Using both known virulence proteins and *in silico* identified surface proteins, our study reports on immunogenic GBS peptides that seem to be involved during bacterial infection and thus further experiments are required to determine their pathogenicity and protective potential. The GBS surface proteins gbs0233 and gbs1539 described here induced higher antibody levels when colonising the vaginal tract of women; the main site associated with vertical transmission of GBS to neonates. These proteins also demonstrated serological potential in preventing GBS acquisition during pregnancy and therefore feasible as candidates for GBS vaccine targets.

## Material and Methods

### Study population

We investigated serum samples from a previously enrolled cohort in which the association between serotype-specific capsular antibodies and GBS recto-vaginal colonisation in pregnant women were investigated^5^. Briefly, the study cohort consisted of 661 asymptomatic pregnant women attending routine antenatal clinic visits in Soweto, South Africa during the period of August 2010 to August 2011. The pregnant women were enrolled at 20–25 weeks of gestation and followed up until to ≥37 weeks of gestational age (prior delivery). The women were confirmed to be HIV-uninfected and not on antibiotic treatment for at least two weeks prior to enrolment. The study population was documented to have a GBS acquisition rate of 25.9% from 20 to ≥37 weeks of gestation age, with 49.6% being colonised at least on one occasion during the study period^5^.

The GBS colonisation status of each study participant was availabe as previously determined at 20–25, 26–30, 31–35, and at ≥37 week of gestation by culturing lower vaginal and rectal swabs^5^. A participant with GBS detected at either the vaginal and/or rectal swab was considered as colonised; and non-colonised if no growth was detected from either swab. In addition, sera were available from the 20–25 weeks and ≥37 weeks visits and was stored at −70 °C until analysed.

Paticipants were categorised as follows: “non-carriers” were women in whom GBS could not be detected at any study visit; and women who were not colonised at visit-1 but colonised at subsequent visits were grouped as “new-acquisition” cases. Women who were colonised with a specific GBS serotype at all study visits were denoted as “persistently colonised” and women who tested positive for GBS at visit 1 (20–25 weeks of gestation age) but had negative culture results at any stage up until the ≥37 weeks gestation age visit were grouped as “transiently colonised”. Pregnant women who were only colonised at either the 2^nd^ or 3^rd^ visit were grouped as “intermittently colonised”.

### Antigens and serological methods

A multiplex-Luminex immunoassay was used to quantify serum IgG titres to GBS surface proteins FbsA, BibA-COH1, BibA-NEM316, Sip, Foldase PsrA, gbs0233, gbs0392, gbs0393, gbs1356, gbs1539 and gbs2106. Proteins FbsA, BibA and gbs0233 were obtained from Valneva Austria GmbH. The remaining protein antigens were *in silico* identified and produced in-house using GBS NEM316 strain as previously described^39^. GBS surface proteins were selected based on their immunogenicity scores as determined using BepiPred software, a bioinformatics tool that predicts linear epitopes on peptides using hidden Markov models and hydrophilicity propensity scales^40^.

Protein antigens were coupled on to microbeads using the 2-step carbodiimide reaction^41^, at a protein concentration of 50 µg/ml per unique bead region. Pooled purified IgG from adult humans (Polygam; National Bioproducts, South Africa) was assigned an arbitrary value of 100 U/mL and was used as a reference serum to measure IgG titres. IgG titres specific for the protein antigens were reported as arbitrary units per millilitre (U/ml) as extrapolated from the Polygam sera. High and low titre sera were pooled from select women and were included in all assays to assess intra-assay reproducibility (≤30% CV). IgG titres for the test samples, controls and reference serum were measured in duplicates at 1:100 dilutions in PBS, 10% fetal bovine serum, 0.05% NaN_3_. Sample analysis was performed using the Bio-plex 200 instrument (Bio-Rad, USA) as previously described^39^.

### Statistical analysis

Statistical analysis was performed using STATA software (version 13.0 Stata-Corp, Tx USA) and GraphPad Prism (version 5.03 GraphPad Software Inc, California USA). Antibody titres remained skewed after log_10_ transformation and therefore median IgG titres are reported. Difference in IgG titres between groups was determined using Mann Whitney *U* test. Spearman’s rank test regression was performed to determine correlation of protein specific IgG titres with maternal age, gestational age, parity and gravidity. Association between colonising serotype and protein specific IgG titres was assessed using Kruskal-Wallis test. Multivariate quantile regression was used to correct for confounding variables with p value < 0.2 when analysed as univariates. χ^2^ and Fisher’s exact test were used to determine association between groups. Differences were considered significant for *p*-value < 0.05.

### Ethics

The study was approved by the Human Research Ethics Committee of the University of the Witwatersrand (Approval number: M121019). All study participants had provided written-informed consent for inclusion into the parent study and methods were carried out as stipulated in the guidelines and regulations.

### Data availability

The data supporting findings presented in this manuscript are available from the corresponding author upon request.

## Electronic supplementary material

Supplementary information
