# Supplementary material for: Association between antibodies against group B Streptococcus surface proteins and recto-vaginal colonisation during pregnancy
Source: Sci Rep. 2017 Nov 28;7:16454. doi: 10.1038/s41598-017-16757-9 (PMC5705700; doi:10.1038/s41598-017-16757-9)
Supplement: Supplementary file 1 — Supplementary information [file 41598_2017_16757_MOESM1_ESM.pdf]

Association between antibodies against group B *Streptococcus* surface proteins and recto-vaginal colonisation during pregnancy

Sonwabile Dzanibe[1,2], Gaurav Kwatra[1,2], Peter V. Adrian[1,2], Sheila Z. Kimaro Mlacha[1,2], Clare L. Cutland[1,2], and Shabir A. Madhi*[1,2,3]

Table S1 Demographic characteristics of pregnant women who were either non-colonised or colonised with group B *Streptococcus* at 20-25 and ≥37 weeks of gestation.

Demographic	20-25 week of gestation		≥37 week of gestation	
	Non-colonised (n=344)	Colonised (n=161)	Non-colonised (n=365)	Colonised (n=140)
Median age, year (IQR)	25.4 (21.7-29.4)	25.5 (22.2-29.7)	25.2 (21.7-29.3)	25.6 (22.2-29.6)
Median parity (range)	0 (0-5)	1 (0-4)	0 (0-5)	1 (0-4)
Median gravidity (range)	2 (1-8)	2 (1-6)	2 (1-8)	2 (1-6)
Miscarriage(s)				
0	293 (85.2%)	128 (79.5%)	304 (82.3%)	117 (83.6%)
1	40 (11.6%)	30 (18.6%)	50 (13.7%)	20 (14.3%)
≥2	11 (3.2%)	3 (1.9%)	11 (3.0%)	3 (2.1%)
Colonisation site				
Rectal†		48 (29.8%)		46 (32.9%)
Vaginal‡		44 (27.3%)		29 (20.7%)
Recto-vaginal		69 (42.9%)		65 (46.4%)
Serotype				
Ia		71 (44.1%)		50 (35.7%)
Ib		7 (4.3%)		7 (5.0%)
II		13 (8.1%)		11 (7.9%)

III	52 (32.3%)	49 (35.0%)
IV	4 (2.5%)	2 (1.4%)
V	13 (8.1%)	17 (12.1%)
IX	1 (0.6%)	4 (2.9%)

† rectal colonisation without concomitant vaginal colonisation, ‡ vaginal colonisation without concomitant rectal colonisation.

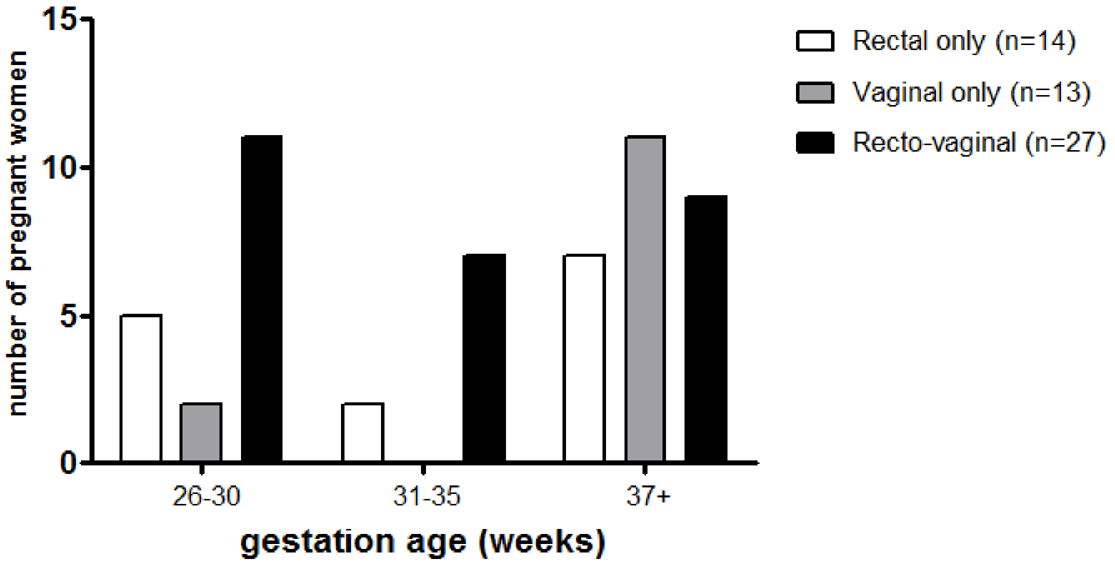

Figure S1 Pregnant women who newly acquired group B *Streptococcus* colonisation after 20-25 weeks gestation age study visit in the rectal tract only (white bars), vaginal tract only (grey bars) and in the rectal and vaginal tract (black bars).

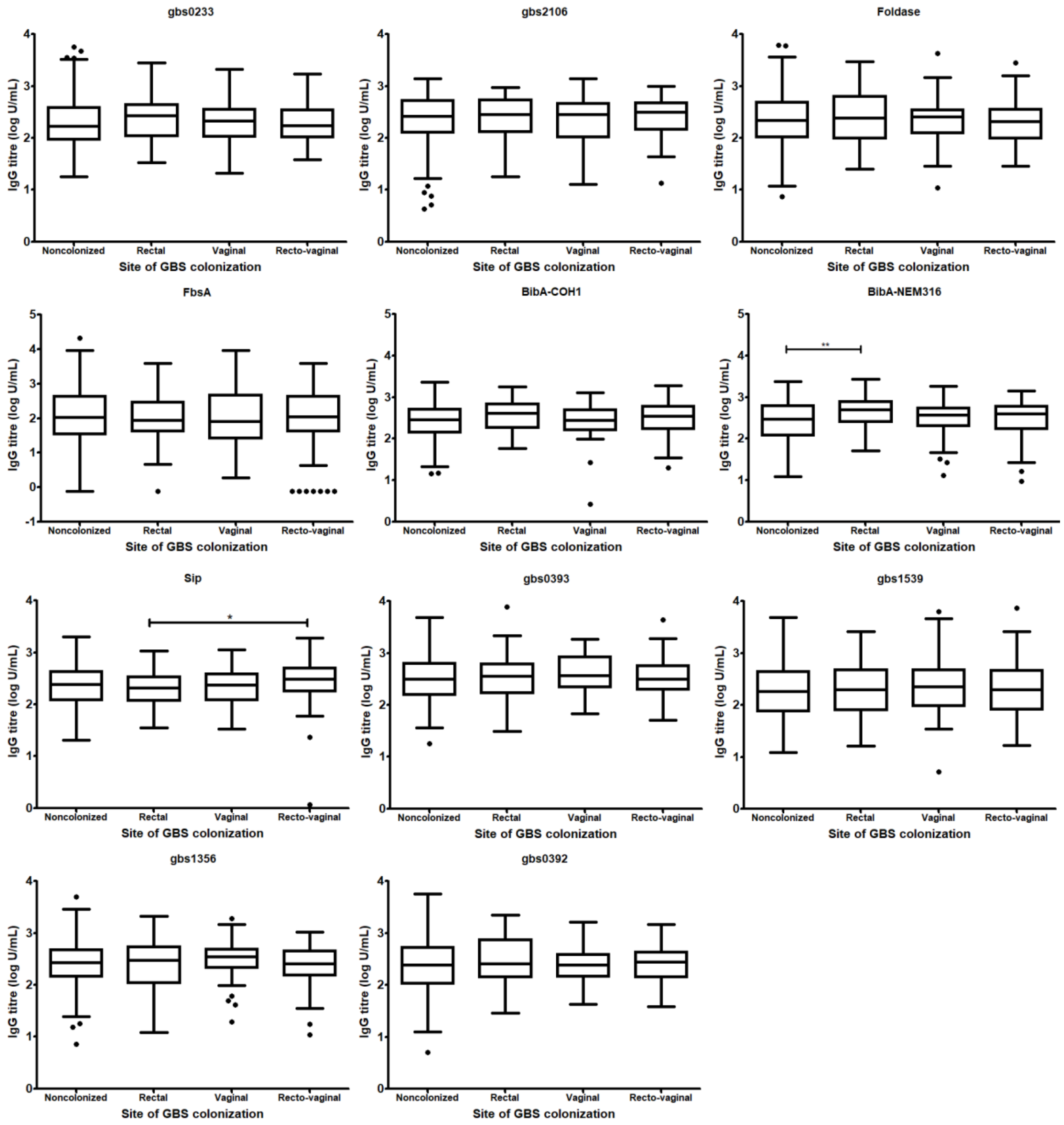

Figure S2 IgG titres (log U/mL) against group B *Streptococcus* surface protein present in pregnant women who were either not colonised or colonised at the rectal and/or vaginal tract at 20-25 weeks of gestation age. Statistical comparison between groups using quantile regression, * $p<0.05$ and** $p<0.01$ adjusted for cofactor variables. Turkey box-and-whisker plots representing the median (line within the box), 25th and 75th percentiles (box), the 1.5 times interquartile distances from the 25th and 75th centiles (whiskers) and outliers (solid dots)

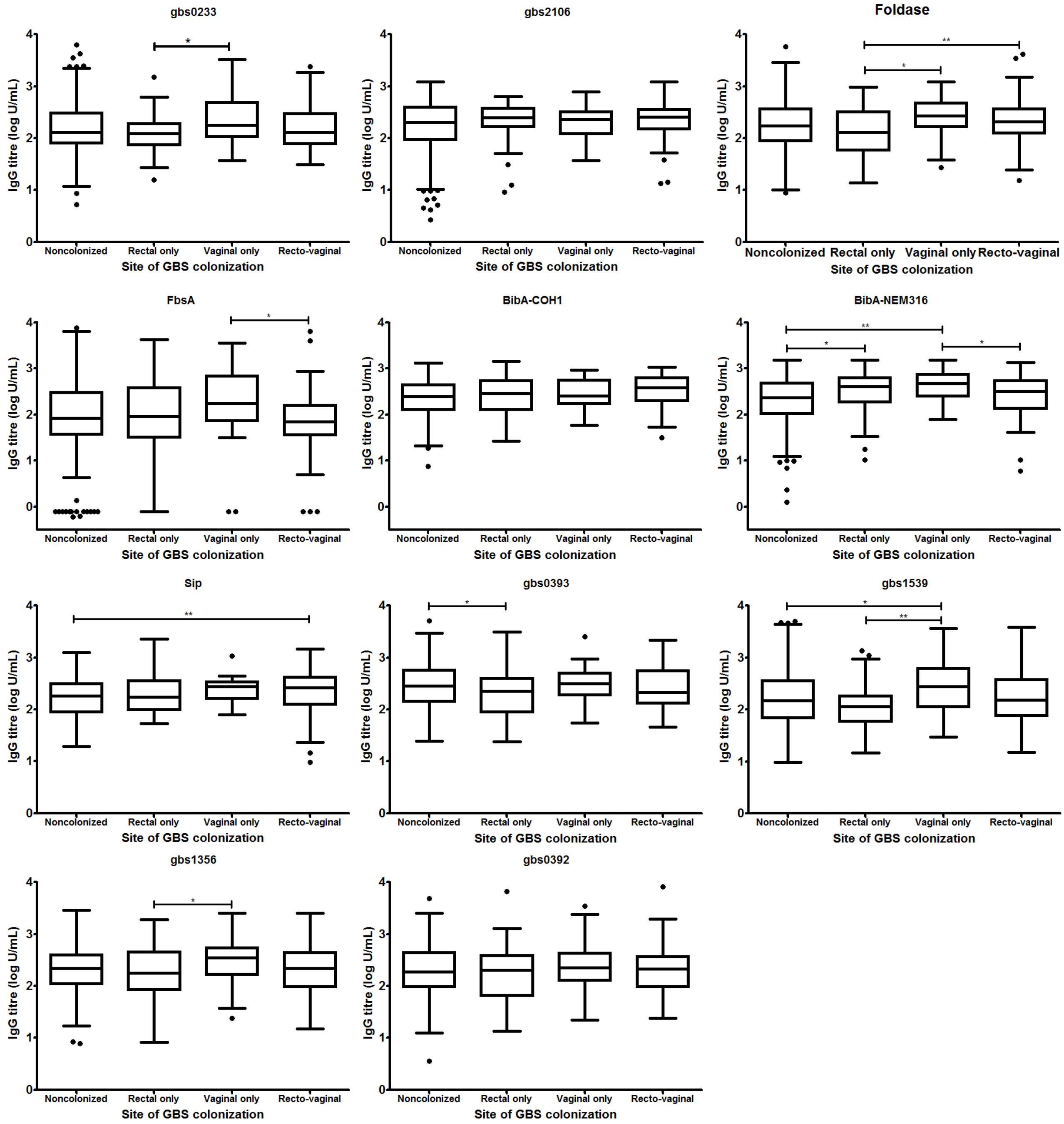

Figure S3 IgG titres (log U/mL) against group B *Streptococcus* surface protein present in pregnant women who were either not colonised or colonised at the rectal and/or vaginal tract at ≥37 weeks of gestation age. Statistical comparison between groups using quantile regression, * p<0.05 and** p<0.01 adjusted for cofactor variables. Turkey box-and-whisker plots representing the median (line within the box), 25th and 75th percentiles (box), the 1.5 times interquartile distances from the 25th and 75th centiles (whiskers) and outliers (solid dots).
